# Invasive Pneumococcal Disease (IPD) Serotype Frequency in Iranian Patients

**DOI:** 10.5812/ircmj.4145

**Published:** 2013-08-05

**Authors:** Ali Mehrabi Tavana, Ramazan Ali Ataee

**Affiliations:** 1 Health Management Research Center, Baqiyatallah University of Medical Sciences, Tehran, IR Iran; 2 Department of Medical Microbiology, Faculty of Medicine, Baqiyatallah University of Medical Sciences, Tehran, IR Iran

**Keywords:** Streptococcus pneumonia, Serotyping, Invasive Pneumococcal Disease (IPD)

## Abstract

**Background:**

*Streptococcuspneumoniae* as a Gram positive diplococcic is a major worldwide causative agent of morbidity and mortality among young children and the aged. In addition, *Streptococcus pneumoniae* is a versatile human pathogen causing infectious disease ranging from mild infection (i.e. otitis media) to life therating pneumonia and meningitidis in many countries

**Objectives:**

The aim of this study was to determine the frequency type of *Streptococcuspneumoniae* in Invasive pneumococcal disease (IPD) in Iranian patients.

**Patients and Methods:**

A total of 135 *Streptococcuspneumoniae* strains were isolated from patients infectious suspected of invasive streptococcal disease. They were subjected to PCR and bacteriological methods. Out of which, 134 strains of *S.* *pneumoniae* were serotyped and confirmed by PCR method. The data were analyzed by SPSS version 17.0.

**Results:**

The results of this study showed some *S.* *pneumoniae* serotypes were found in both sexes and some only in one sex invasive infections. For example, serotypes 10, 14, 18 and 22 were only in female patients with infections.

**Conclusions:**

The analysis of the results had suggested that serotypes 6 from Lung and 19 from Eye are the most abundant bacterial strains isolated from patients. The diseases could be prevented by using the Pneumococcal vaccine.

## 1. Background

*Streptococcuspneumoniae* as a Gram positive diplococcic is a major worldwide causative agent of morbidity and mortality among young children and the aged (1). In addition, *Streptococcuspneumoniae* is a versatile human pathogen causing infectious disease ranging from mild infection (i.e. otitis media) to life therating pneumonia and meningitidis in many countries (2). The most prevalence of Invasive pneumococcal disease caused by *Streptococcus pneumoniae* is acute Otitis Media, Pneumonia and Meningitis (3). Among this Pneumonia is the main infection with immdeate cure needed for patients (4). It has to be said that Pneumococcal account for most cases of community-acquired (5). The research reports that, 92 capsular serotypes of *Streptococcus pneumoniae* differ greatly in nasopharyngeal carriage prevalence, invasiveness, and disease incidence (6). Therefore, there has been some necessitate, though, regarding whether serotype independently affects the outcome of invasive pneumococcal disease (IPD).

## 2. Objectives

The aim of this study was to determine the frequency type of Streptococcus pneumoniae in Invasive *pneumococcal* disease (IPD) in Iranian patients.

## 3. Materials and Methods

The present study was conducted on as a prospective study to type the profile of pneumococcal strains from Invasive Pneumococcal Disease (IPD) across in Iran. During two years (2009 – 2010) all invasive Pneumococcal strains isolates from selected laboratory hospitals in Iranian central provinces were collected. The strains were isolate from culture of throat, Blood, Sputum, CSF were fluid; eye and lung infection. They were transfer to central Baqiyatallah laboratory and re- identified based on bacteriological standard methods. A total of 135 bacteria strain suspected of invasive streptococcal disease (IPD) were subjected to PCR, bacteriological methods and serotyped. All isolates were serotyped by the Quellung reaction using specific antiserum (Statens Serum Institute, Copenhagen, Denmark) based on manufacture procedure. Out of which, 134 isolated strains of *S. pneumoniae* was confirmed by PCR method.

### 3.1. Laboratory Investigation

The isolated invasive streptococcal strains were re- identified as pneumococcal according to Standardized Procedure to culture and analyze (-hemolysis, colony morphology, optochin susceptibility, and bile solubility) (7). The samples have been tested on the Blood agar and Optochin sensivity and bile solubility as well as Gram Staining was performed too. The positive results were noted. A reaction in which anticapsular antibodies bind to the capsule of a bacterium, resulting in the capsule to swell or become more visible, was done and it was seen under the microscope. The pneumococcal isolates were then typed by immunological techniques according to Company Procedure (SSI. Denmark) (8). The demographic data including (age, sex, site of infection) was accumulated. The data was inserted in the computer and then specific Pneumococcal types were matched and then collected data analyzed by SSPS version 17.0.

## 4. Results

**Figure 1.**
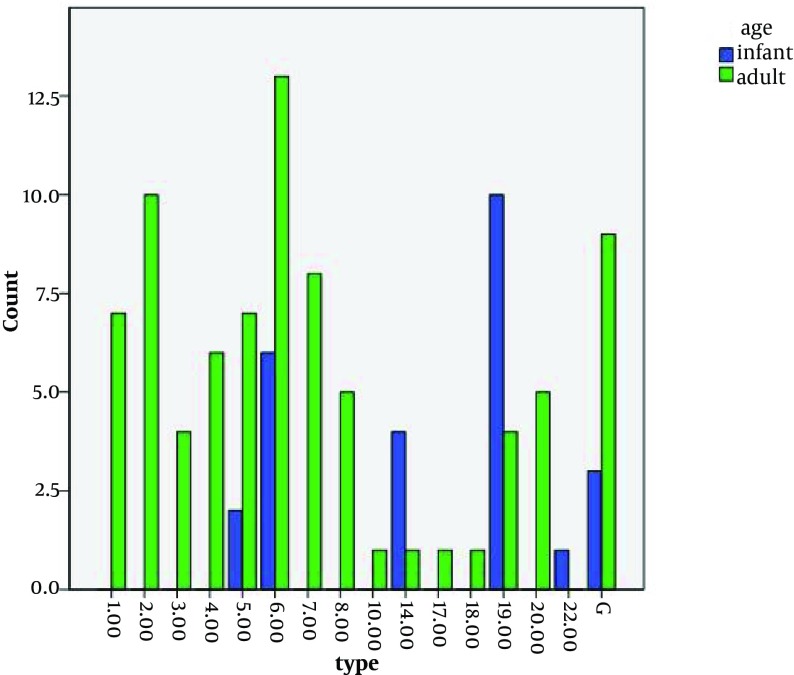
Types of S. pneumoniae and Age of Patients

**Figure 2.**
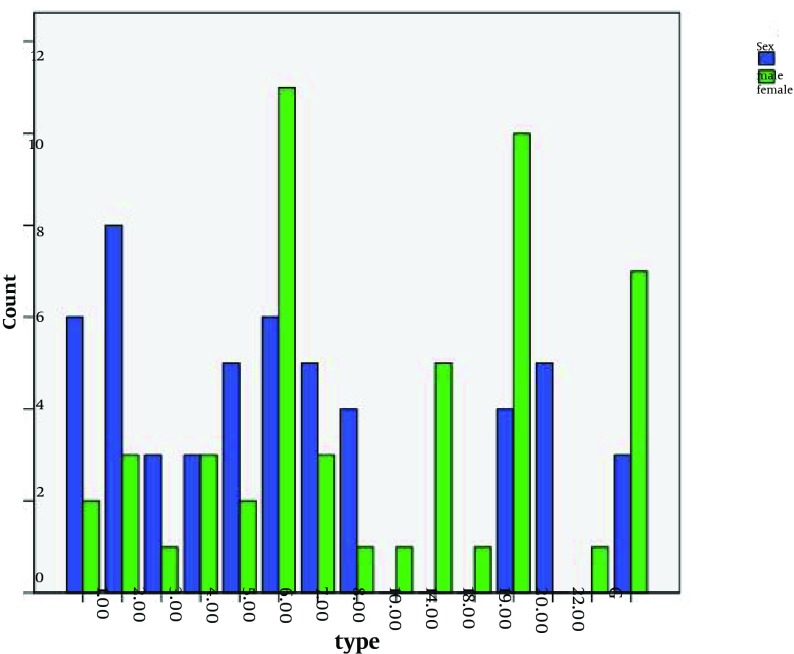
Types of S. pneumoniae and Sex of Patients

The results of this study showed some *S.pneumoniae *serotypes were found in both sexes and some only in one sex invasive infections. For example, serotypes 10, 14, 18 and 22 were only in female patients with infections. While serotypes 20 were only isolated from men infected. The analysis of the results had suggested that serotypes 6 and 19 are the most abundant bacterial strains isolated from patients. (See Figure 1 and Figure 2). The analysis results of serotype distribution based on site of infections were showed in Table 1. 

**Table 1.** Serotype Distribution and Site of Isolation of Invasive Streptococcal Strains

Site of Isolation	Number of Frequecy	Serotype or Serogrope
**Wounds**	3	6
	3	5
	2	10
**Rhinit**	4	7
**Sinus**	3	20
**Eye**	9	6
	4	1
	2	14
	11	19
**Throat**	3	19
	3	7
	3	22
	5	20
	6	14
	4	3
	5	4
	3	1
**Lung**	2	1
	5	2
	3	7
	3	G
	4	5
	9	6
	5	8
	3	4
	3	17
	2	18
**blood**	1	17
	5	G
	1	1
	5	2
	3	6
**CSF**	3	20
	3	8

## 5. Discussions

Our data is different from other research. For example, some studies were shown that, the serotypes 1 and 5 were commonly causing IPD ( 9 - 13 ). While in this study, the serotypes 6 and 19 were the most common involved in invasive streptococcal infections (Table 1). Recently, in national level the results of a report was published has been shown similar data. They were serotyped only 75 *S. pneumoniae *strains and due to limited sample have claimed that heptavalent pneumococcal conjugate vaccine (PCV7) are covering 80% of the serotypes ( 14 ). Based on the results of this study, the determine serotypes involved in invasive infections are equal the 23 valent pneumococcal vaccine (PPV23). However, the compare data but a few studies may be similar typing results in different country. In Denmark overall, 92% (93% blood, 87% CSF) of isolates and 94% of all childhood isolates belonged to the 23 vaccine types. In addition, In Denmark, the ten most frequently occurring types from children were (6A + 6B, 18C, 14, 1, 7F, 19F, 9V, 4, and 23F) ( 15 ). Pneumococcal types may be different country by country for this reason different vaccine (Heptavalent Pneumococcal Conjugate Vaccine, 7 valant or 13-valent pneumococcal conjugate vaccine or even 23 valents has been applied in different countries in order to prevent the disease in different age groups ( 16 - 18 ). Hence, the only vaccine that is able coverage serotypes our country is the 23-valent vaccine. Molecular typing must be applied in the further me be helped the details ( 19 ). 
